# Profil épidémiologique et clinique de la tuberculose dans la zone de santé de Lubumbashi (RD Congo)

**DOI:** 10.11604/pamj.2014.17.70.2445

**Published:** 2014-01-28

**Authors:** Christian Kakisingi Ngama, Michel Manika Muteya, Yves Isango Idi Lukusha, Serge Matanda Kapend, Henri Mundongo Tshamba, Paul Ilunga Makinko, Claude Mwamba Mulumba, Liévin Kapend a Kalala

**Affiliations:** 1 Service de médecine interne, Cliniques Universitaires de Lubumbashi, Unilu, BP 1825, RD Congo; 2 Service d'anesthésie-réanimation, Cliniques Universitaires de Lubumbashi, Unilu, BP 1825, RD Congo; 3 Service de Gynéco-obstétrique, Cliniques Universitaires de Lubumbashi, Unilu BP 1852, RD Congo; 4 Service de Laboratoire, Cliniques Universitaires de Lubumbashi, Unilu, BP 1825, RD Congo; 5 Médecin chef zone de santé de Lubumbashi, District de santé de Lubumbashi, Katanga, RD Congo; 6 Ecole de Santé Publique, Université de Lubumbashi(Unilu), BP 1825, RD Congo; 7 Service de Pédiatrie, Cliniques Universitaires de Lubumbashi, Unilu, BP 1825, RD Congo

**Keywords:** Tuberculose, zone de santé, Lubumbashi, Tuberculosis, Health Zone, Lubumbashi

## Abstract

**Introduction:**

L'objectif de notre travail était de déterminer la distribution sociodémographique des patients tuberculeux, les types de tuberculose en fonction de la localisation de la maladie et déterminer l'issue thérapeutique des patients en fonction de différentes localisations.

**Méthodes:**

C'est une étude descriptive transversale des patients diagnostiqués et traités pour tuberculose du 1^er^ Janvier 2010 au 30 Juin 2011 dans la zone de santé de Lubumbashi. Une de 11 zones de santé du District de Lubumbashi dans la province du Katanga(RD Congo). Ont été inclus tous les patients tuberculeux de nationalité congolaise consultés dans la zone de santé pendant la période d’étude. L’âge, le sexe, la commune de résidence, le tableau clinique à la première consultation et les résultats des examens de laboratoire des crachats par la coloration Ziehl-Neelsen ont été les paramètres d'analyse.

**Résultats:**

Nous avons enregistré 708 patients tuberculeux soit une prévalence de 0.5%. Le sexe masculin représentait 58.78% contre 41.25% de sexe féminin avec un sexe ratio de 1.42 en faveur du sexe masculin. La moyenne d’âge était de 33±;15 ans. La majorité des patients soit 54.79 appartiennent à la tranche d’âge entre 21 et 40 ans. La tuberculose extra pulmonaire a représenté 51.8% contre 50.2% de tuberculose pulmonaire dont 31.9% à bacilloscopie positive. Le décès a intéressé les patients bacillifères puis qu'il y a 5 fois plus de décès liés à une tuberculose pulmonaire à microscopie positive qu'aux autres formes de tuberculose (OR (IC 95%): 5.27 (2.92-9.59, p = 0.00). La majorité des patients résidaient les communes Lubumbashi (41.7%) et Kampemba (23.2%).

**Conclusion:**

La tuberculose extrapulmonaire (pleurale) a été plus rencontrée que la tuberculose pulmonaire et c'est cette dernière forme de tuberculose qui a entrainé beaucoup plus de décès. Ce qui nécessite une amélioration du système de santé de prise en charge des tuberculeux dans les démarches diagnostique, le suivi des patients bacilliformes et encourager l'adhérence au traitement.

## Introduction

La tuberculose est une maladie encore largement répandue en Afrique subsaharienne où les mesures visant à freiner son expansion souffrent souvent de faiblesses liées aux contraintes financières, au niveau de vie de la population et au système de santé en place [1, 2].

En RDC, la lutte contre la tuberculose est coordonnée par le programme national de lutte contre la tuberculose (PNLT). Ce programme a intégré un protocole de prise en charge dans les centres de santé de dépistage et de traitement de la tuberculose (CSDT) réunissant théoriquement un équipement minimum pour répondre aux besoins de la population dans cette matière^3^ Depuis 2008, le protocole de prise en charge a intégré le dépistage systématique de l'infection à VIH. Ainsi, une étude épidémiologique régulière permet une bonne évaluation du suivi des activités de prise en charge de la tuberculose.

Au niveau de la coordination Provinciale de lutte contre la tuberculose Katanga Sud, l'incidence de la tuberculose est estimée à 0.18% pour une population estimée à 3 636 747 habitants en 2010 et le taux de décès chez les nouveaux cas de tuberculose à microscopie positive a été estimé à 5% pour 3687 cas enregistrés au cours de la même année [3].

L'objectif de ce travail est de déterminer la distribution sociodémographique des cas de tuberculose, la distribution des types de tuberculose en fonction des localisations et de l'issue thérapeutique des patients en fonction des localisations rencontrées.

## Méthodes

### Type et période d’étude

Il s'agit d'une étude descriptive transversale des patients diagnostiqués et traité pour tuberculose réalisée du 1^er^ Janvier 2010 au 30 Juin 2011 dans la zone de santé de Lubumbashi, une de 11 zones de santé du district de Lubumbashi, dans la province du Katanga(RD Congo). Elle compte 3 centre de santé de dépistage et traitement de la tuberculose(CSDT) à savoir les Cliniques Universitaires de Lubumbashi, l'Hôpital Provincial de référence Jason Sendwe et le Centre Médical du Centre Ville(CMDC)

### Population d’étude

Ont été inclus tous les patients tuberculeux de nationalité congolaise consultés dans la zone de santé pendant la période d’étude.

Les paramètres de l’étude sont: l’âge, le sexe, la commune de résidence, le tableau clinique à la premier consultation précisant la localisation de la pathologie et le résultats des examens de laboratoire des crachats par la coloration Ziehl-Neelsen pour faire la différence entre une tuberculose à microscopie positive et négative [4].

Pour la tuberculose pleurale et/ou péritonéale, les données d'analyse chimique et cytologique des liquides obtenus par ponction ainsi qu'un traitement conclu par une bonne réponse clinique ont permis de retenir le diagnostic. Il en est de même pour la tuberculose ganglionnaire dont le diagnostic était retenu sur base du tableau clinique marqué par des adénopathies chroniques, du bilan biologique et d'un traitement antituberculeux concluant [3]. Dans le même ordre, les résultats du test de dépistage rapide “Determine” pour le VIH ont été recueillis ainsi que le résultat du test de confirmation de la séropositivité à l'ELISA.

### Ethique

Pour des raisons d’éthiques et de déontologie et pour ne pas stigmatiser les centres de santé de diagnostic et de traitement de la tuberculose(CSDT), les résultats n'ont pas été donnés par CSDT et le traitement des données s'est fait de façon anonyme pour tous les patients après avoir recueillis leur consentement.

### Collecte et analyse

Les données ont été encodées et analysées à l'aide des logiciels Epi Info^®^ 3.5.1 et Excel 2007. Statistiquement nous avons eu recours à la moyenne, l’écart-type, au test de Khi-carré non corrigé de pearson et à l'Odd-ratio avec un seuil de signification à 95% et une valeur de p < 0.005.

## Résultats

### Prévalence et répartition en fonction de l’âge et du sexe

**Figure 1:**
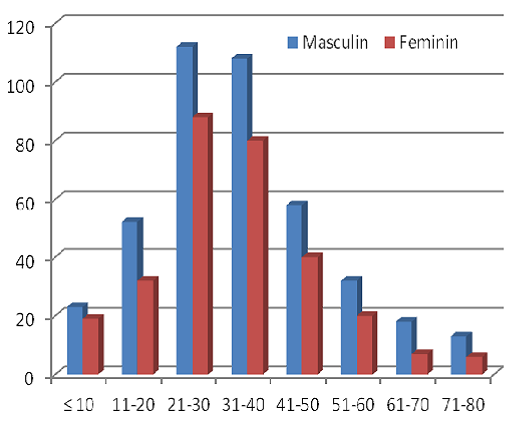
Répartition des sujets tuberculeux en fonction de l’âge et du sexe

Pour une population de 134 809 habitants, 708 cas de tuberculose ont été enregistrés soit une prévalence de 0.5%. Par ailleurs la répartition des patients en fonction de l’âge et du sexe montre que les malades de sexe masculin étaient plus touchés (58.75%) que ceux de sexe féminin (41.25%) avec un sexe ratio de 1,42 en faveur des hommes. L’âge moyen de l'ensemble était de 33 ans ± 15. La majorité des patients tuberculeux font partie de la tranche d’âge entre 21 et 40 ans soit 54.79% (Figure 1).

### Le lieu d'habitation

La majeure partie de ces 708 sujets concernés par notre étude viennent principalement de la commune de Lubumbashi (41.7%) et de celle de Kampemba (23.2%) (Tableau 1).

**Tableau 1:** Répartition des patients en fonction de la commune de provenance

Commune de provenance	n	%
Annexe	63	8.90%
Kamalondo	21	3.00%
Kampemba	164	23.20%
Katuba	83	11.70%
Kenya	33	4.70%
Lubumbashi	295	41.70%
Ruashi	49	6.90%
Total	708	100.00%

### Données cliniques

L'analyse des dossiers de 708 patients a montré qu'il y avait presqu'autant de localisation extra pulmonaire (51.8%) que pulmonaire (50.2%) parmi lesquels 226 (31.9%) étaient à bacilloscopie positive (Tableau 2, Tableau 3). Nous avons enregistré plus de cas de décès dans le groupe de patients bacillifères et nous avons constaté qu'il y a 5 fois plus des décès liés à une tuberculose pulmonaire à microscopie positive(TPM + ) qu'aux autres formes de tuberculose (OR (IC 95%): 5.27 (2.92-9.59, p = 0.00) (Tableau 4).

**Tableau 2:** Répartition des patients en fonction de type et localisation de la tuberculose

Type	Localisation	n	%
TEP^*^	Ganglionnaire	36	5.10%
	Osseuse	28	4.00%
	Péricardique	4	0.60%
	Péritoneale	40	5.60%
	Pleurale	245	34.60%
TP^*^	TPM-	115	16.20%
	TPM +	226	31.90%
**Total**		708	100.00%

TEP = Tuberculose Extrapulmonaire ; TPM- = Tuberculose Pulmonaire à Microscopie Négative ; TPM+ = Tuberculose Pulmonaire à Microscopie Positive ; TP= Tuberculose Pulmonaire.

**Tableau 3:** Répartition des patients en fonction de l'issue thérapeutique

Issue thérapeutique			TEP		TPM-		TPM +	
	n	%	n	%	n	%	n	%
Décès	62	8.75	11	2.99	9	1.27	42	18.58
Echec thérapeutique	7	0.99	0	0	0	0	7	3.10
Guérison	148	20.90	0	0	0	0	148	65.49
Interruption de traitement	12	1.69	1	0.27	1	0.86	10	4.42
Traitement achevé	473	66.80	353	96.18	104	90.43	16	7.08
Transfert out	6	0.85	2	0.54	1	0.86	3	1.32
**Total**	708	100	367	51	115	16.24	226	31.9

TEP = Tuberculose Extrapulmonaire ; TPM- = Tuberculose Pulmonaire à Microscopie Négative ; TPM+ = Tuberculose Pulmonaire à Microscopie Positive ; TP= Tuberculose Pulmonaire

**Tableau 4:** Rapport entre la forme de tuberculose et le décès

Forme de tuberculose	Décès	Survie	Total
TPM +	42	184	226
TPM- et TEP	20	462	482
**Total**	62	646	708

**Khi carré = 40.12 et p = 0.00** ; TEP = Tuberculose Extrapulmonaire ; TPM- = Tuberculose Pulmonaire à Microscopie Négative ; TPM+ = Tuberculose Pulmonaire à Microscopie Positive.

## Discussion

Notre étude revêt un intérêt particulier en ce sens qu'elle permet de nous donner un aperçu sur le fonctionnement des centres de santé de dépistage et de traitement de la Zone de Santé de Lubumbashi. Elle a porté sur 708 cas de tuberculose enregistré dans ces centres de dépistage et de traitement.

Au cours de la période d’étude, une prévalence de 530 cas de tuberculose pour 100 000 habitants a été notée dans la Zone de Santé de Lubumbashi. Cette prevalence semble confirmer la position de la République Démocratique du Congo où celle-ci est placée dans la zone à 300 et plus de cas pour 100 000 habitants [2] et est sensiblement supérieure aux constats observés par d'autres auteurs [5, 6]. Ceci serait le reflet probable de la gravité de la maladie dans notre zone de santé mais ce taux élevé pourrait être lié à une amélioration du dispositif de surveillance.

Dans notre étude, les hommes (58.75%) sont plus affectés que les femmes (41.25%). Nos résultats sont identiques à ceux d'autres études [5–8] effectuées dans certains pays du monde et une analyse des modes de vie pourrait être évoquée face aux conditions de travail difficile dans un contexte de pauvreté généralisée pourrait être un élément d'explication. Notons que les travaux des ménageres, moins exposées aux conditions difficiles en dehors du domicile, seraient un facteur de protection [2].

Les patients tuberculeux enregistrés dans les CSDT de la Zone de Santé de Lubumbashi avaient un âge moyen de 33 ans ± 15 et les tranches d’âge les plus atteintes étaient celles de 21-30 ans (28.24%) et 31-40 ans (26.55%). Mtiraoui et al. ont fait le même constat que le notre au cours d'une étude effectuée dans la région sanitaire de Sousse en Tunisie [5].

Le protocole national de prise en charge de la tuberculose recommande que les CSDT couvrent toute la juridiction de la zone de santé pour une facilité d'accès [3]. Les CSDT de la Zone de Santé de Lubumbashi ont été fréquentés par les habitants venant de toute la ville de Lubumbashi mais la fréquentation des habitants provenant des communes de Lubumbashi (41.70%) et Kampemba (23.20%) ont été les plus importantes. L'accès des patients à ces centres a été certainement favorisé par la proximité, mais aussi par la facilité de circulation vers ces centres et la fonctionnalité des structures de prise en charge situées au centre de la ville, site à convergence vers les activités commerciales, activités de survie, distractives et politiques.

La forme clinique de la tuberculose est dominée par la localisation extrapulmonaire (51.8%) et l'atteinte pleurale (34.6%) est la forme clinique des tuberculoses extrapulmonaires la plus fréquente. Bien que, l'atteinte pleurale soit la forme la plus fréquente des atteintes extrapulmonaires de la tuberculose, comme l'ont aussi constaté Baroux et D'Ortenzio dans leurs études faites dans l’île de la Réunion^8^, notre constat par rapport à la forme clinique la plus fréquente diffère de celui obtenu dans la région sanitaire de Sousse, en France et l’île de la Réunion où la forme pulmonaire est la plus fréquente dans les proportions respectives de 66%, 71.1% et 81%^5^, ^7^, ^8^].

Le taux de mortalité lié à la tuberculose est estimé à 8.75% dans notre étude et ce taux est légèrement inférieur à ceux trouvé par Eholie et Al. et Daucourt et al [9, 10] qui sont respectivement de 11% et 14%. Les facteurs de risque de mortalité n'ont pas fait l'objet de notre etude mais nous pouvons néanmoins incriminer les localisations multiples de la tuberculose [11] et l'existence d'une multirésistance[12, 13]. La plus forte mortalité est retrouvée chez les patients ayant une tuberculose pulmonaire à microscopie positive (18.58%). Cette forme clinique reste la plus meurtrière dans notre observation et le risque de décès est cinq fois plus élevé quand on souffre de tuberculose pulmonaire confirmée par microscopie que d'autres formes de tuberculoses réunies.

## Conclusion

Cette étude sur la tuberculose dans la zone de santé de Lubumbashi, nous a permis de noter que l'incidence de cette maladie est de 530 cas de tuberculose pour 100 000 habitants et que le sexe masculin est le plus concerné. La localisation pleurale a été la forme la plus rencontrée parmi les formes extrapulmonaire et la forme pulmonaire à microscopie positive reste celle qui a le taux de mortalité le plus élevé. Nous estimons que le système de santé pour la prise en charge des patients tuberculeux est encore à améliorer dans l'ensemble de cette portion de la ville de Lubumbashi pour espérer une réduction de la morbi-mortalité liée à la tuberculose.
